# Case Report: Rare Iliac Vein Compression (May–Thurner) Syndrome in a Pediatric Patient

**DOI:** 10.3389/fped.2021.694782

**Published:** 2021-07-15

**Authors:** Lianfu Ji, Fan Yang, Xuan Chen, Jinlong Chen, Xueying Cheng, Jie Yin, Yuming Qin, Shiwei Yang

**Affiliations:** Department of Cardiology, Children's Hospital of Nanjing Medical University, Nanjing, China

**Keywords:** iliac vein compression syndrome, iliac vein, iliac artery, left lower extremity swelling, angiography

## Abstract

Iliac vein compression syndrome (IVCS) or May–Thurner syndrome occurs predominantly in young to middle-aged women. Here we reported a case of IVCS in a 5-year-old boy. The child was admitted to our vasculocardiology department with left lower extremity that had been swollen for 1 month. Blood tests revealed coagulation routine and platelets in the normal ranges. Computer tomography angiography (CTA) and magnetic resonance imaging (MRI) showed the left common iliac vein had become narrow before it entered the right common iliac vein. To further clarify, we performed angiography, which clearly showed the stenosis and the blood return of the left common iliac vein. So IVCS was diagnosed. What is more, we found the aorta descended to the right of the spine, and this may be the reason for the apparent compression of the left common iliac vein. Given the young age and mild symptoms of the child, the treatment was conservative mainly including elevation of the affected limb, wearing medical elastic socks, and short-term oral aspirin for anticoagulation. Meanwhile, the boy is being followed up closely. If the swelling of the left lower extremity significantly increases, stent placement may need to be considered in the future.

## Introduction

Iliac vein compression syndrome (IVCS) is a common anatomic variation of the narrowing of the left common iliac vein across the right common iliac artery and the lumbar vertebral body, causing lower limbs and pelvic venous reflux disorder; this is also called Cockett syndrome or May–Thurner syndrome (1). In addition to the anatomical phenomenon of compression of the left common iliac vein, it has been reported that the right common iliac vein was compressed by the right common iliac artery (2). The main clinical symptoms of IVCS include lower limb swelling, pain, varicose veins, pigmentation, deep vein thrombosis, chronic venous ulcer, and claudication. It can also lead to serious complications such as pulmonary embolism (3).

Symptomatic IVCS occurs predominantly in women aged 20–40 years old (4). However, the IVCS often goes unrecognized in most cases, especially in children. A small amount of relevant literature based on children has been reported, not to mention younger ones relatively. Here, we report a case of IVCS in a 5-year-old boy whose left lower extremity was swollen for 1 month.

## Clinical presentation

**Figure 1:**
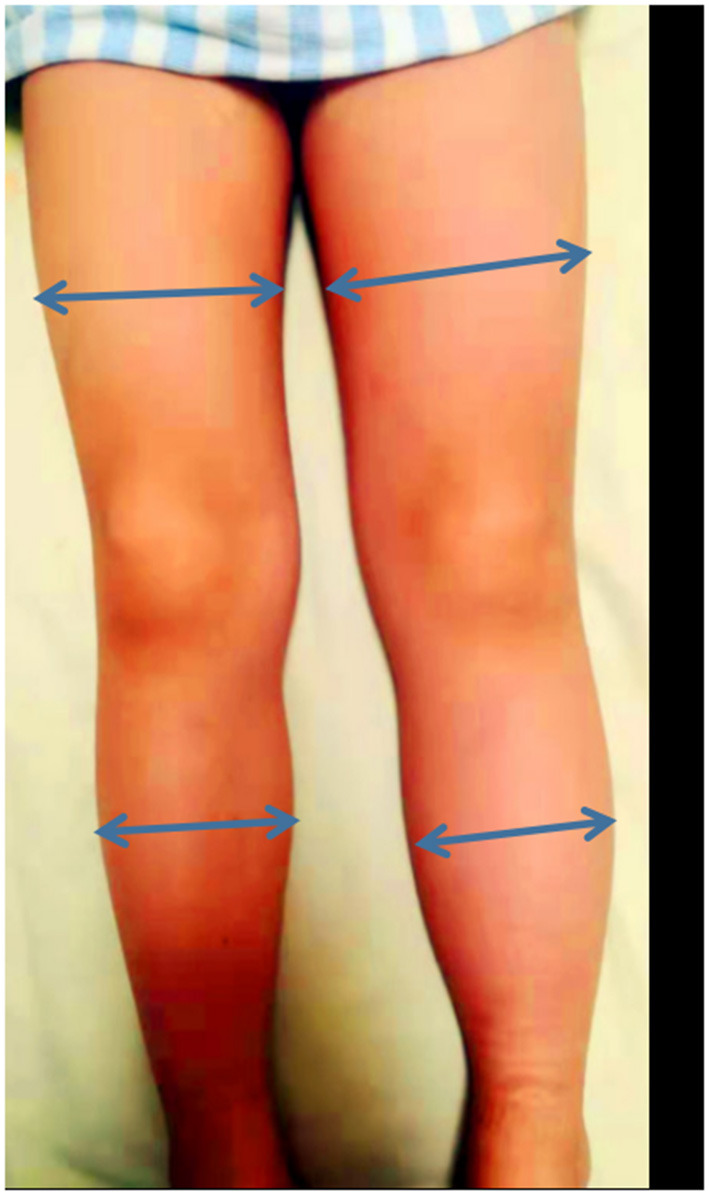
The left lower extremity swelling.

A 5-year-old boy was admitted to our vasculocardiology department with his left lower extremity swollen for 1 month. The boy had no obvious pain, varicose veins, pigmentation, or limitation of activity. We measured the circumference of the thigh and calf at their midpoints. It was 29 and 24 cm on the not-normal left side and 27 and 22 cm on the normal right side (see Figure 1). He had no history of surgery, long travel, or infection. On admission, blood tests revealed coagulation routine and platelets in the normal ranges.

**Figure 2:**
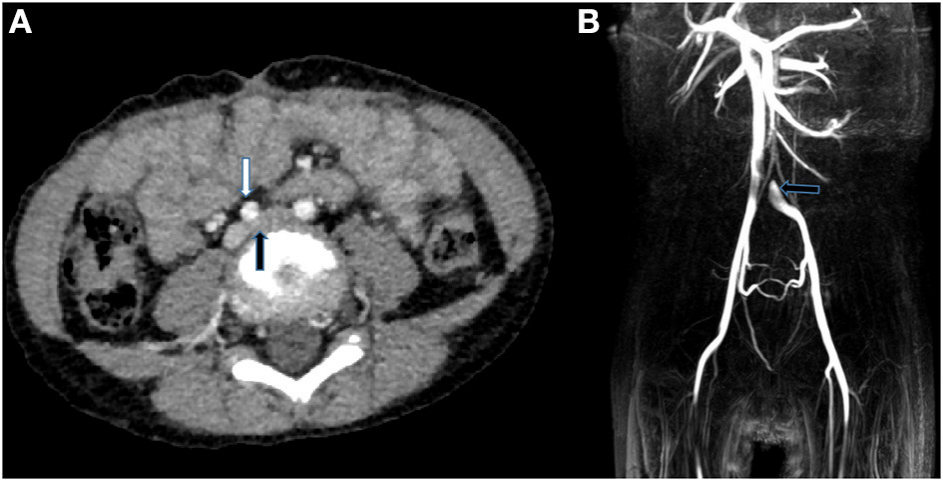
**(A)** CTA in the transverse plane showed the left common iliac vein (black arrows) was compressed between the right common iliac artery (white arrow) and the lumbar vertebral body. **(B)** MRI images indicated that there was filling defect where the left common iliac vein merged into the right common iliac vein.

**Figure 3:**
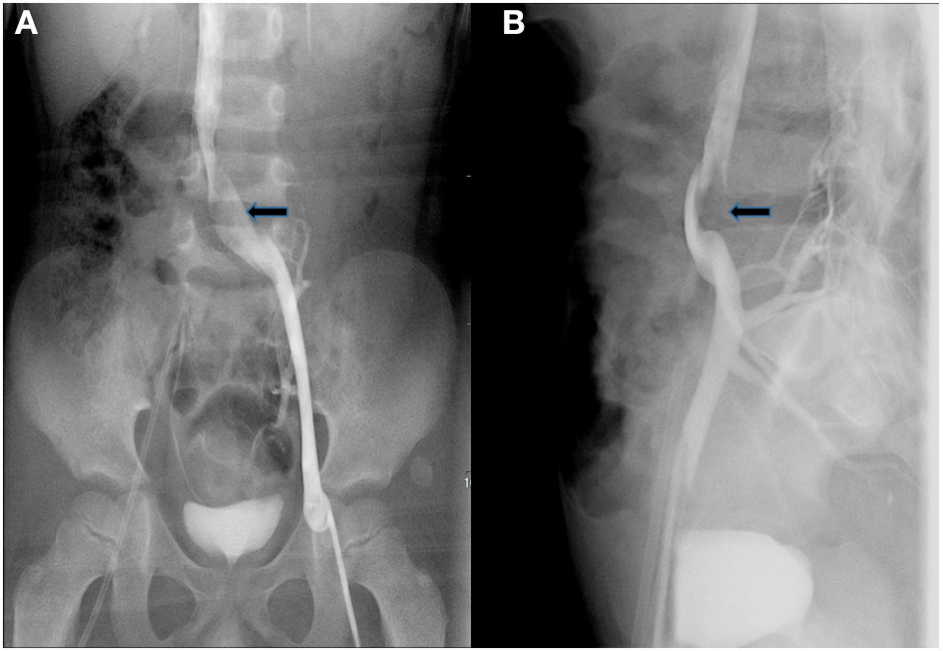
Anteroposterior **(A)** and lateral **(B)** angiography showed that the anterior end of the narrow left common iliac vein was dilated and had filling defect (black arrows).

**Figure 4:**
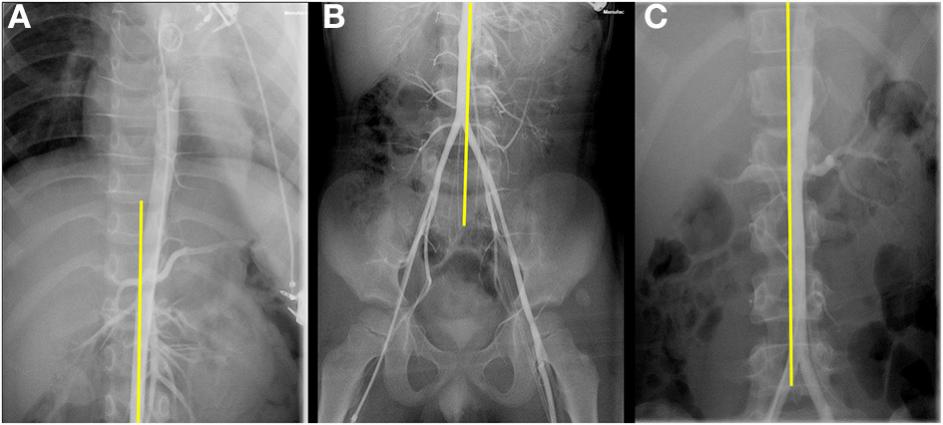
**(A,B)** The aorta descended diagonally on the right side along the middle of the spine. **(C)** Aortogram of a boy suspected of renal artery stenosis used as a control.

Next, we did imaging examinations. Color Doppler sonography of the limbs showed no abnormalities in the main arteries and associated veins in both lower limbs and no thrombosis. Computer tomography angiography (CTA) showed no obvious abnormality in the whole aorta, but the images in the transverse plane showed that the left common iliac vein was compressed between the right common iliac artery and the lumbar vertebral body (see Figure 2A). Magnetic resonance imaging (MRI) confirmed the presence of compression (see Figure 2B). To further clarify, we performed angiography. The angiography process was as follows: First, angiography of the left and right femoral vein was performed: the anterior and posterior diameters of the left common iliac vein were narrowed, and the left and right diameters were broadened where the left common iliac vein merged into the right common iliac vein; the anterior end of the narrow left common iliac vein was dilated and had filling defect (see Figure 3). In addition, the thoracic aorta, abdominal aorta, and femoral artery were examined: the aorta was found to be abnormal at the diaphragmatic level, descending diagonally on the right side along the middle of the spine (see Figure 4). The blood flow of the aorta and its branches were normal, and there was no filling defect.

## Discussion

In 1851, Virchow discovered that the left lower extremity is more prone to thrombosis than the right lower extremity. In 1957 May and Thurner examined 430 cadavers and found 22% of the studied subjects have “spur” formation where the right common iliac artery crossed the left common iliac vein (1, 4). The iliac vein being subjected to mechanical compression of the crossing iliac artery and the influence of artery pulsation on the wall of the vein can easily lead to vein wall thickening, wall injury, and venous thrombosis.

In fact, iliac vein compression is a common anatomic variation. Many people have varying degrees of compression of the left common iliac vein but have no clinical manifestations. So the presence of venous symptoms is critical to IVCS diagnosis (5, 6). IVCS mainly occurs in young and middle-aged women, and 18% to 49% of patients manifested deep venous thrombosis in the left lower extremity (7). Both iliac vein compression itself and iliofemoral vein thrombosis can lead to chronic venous insufficiency in the lower extremity manifested by lower extremity swelling, pain, varicose veins, skin pigmentation, ulcers or recurrent superficial phlebitis, intermittent claudication, and other symptoms (8, 9).

Color Doppler ultrasound, computed tomography venography (CTV), and magnetic resonance venogram (MRV) can assist in the diagnosis of iliac vein compression. They are relatively noninvasive methods. Color Doppler ultrasound is the simplest and cheapest imaging examination, but the sensitivity is low (10). With the development of imaging technology, more and more attention has been paid to the value of CTV and MRV in the diagnosis of iliac vein compression. They can clearly visualize the presence of compression and stenosis of the left common iliac vein (11). Until now, angiography has been regarded as the gold standard for the diagnosis of venous disease. The main angiographic findings of IVCS are as follows: (1) the wider diameter of the left common iliac vein at the compression segment; (2) filling defect of the compression segment of the left common iliac vein; (3) left common iliac vein occlusion; (4) collateral circulation form; and (5) delayed venous emptying of affected limbs.

At present, the main treatment methods for IVCS include surgical treatment and non-surgical treatment. Traditional treatment regimens are mainly for lower extremity deep vein thrombosis and venous dysfunction caused by iliac vein compression, mainly including elevating the affected limb and wearing elastic socks. For iliac vein thrombosis caused by iliac vein compression, the traditional treatment method is mainly oral warfarin anticoagulation therapy after heparin treatment (12). Because non-surgical treatment cannot relieve the underlying cause of iliac vein compression and the thrombosis can easily occur repeatedly, the clinical symptoms are further aggravated. In recent years, endovascular therapy (including balloon dilation and stent implantation) has gradually become a research hotspot in the treatment of IVCS. Numerous studies in adults have shown that the short-term efficacy of stent implantation for IVCS is satisfactory, and the patency rate of mid- and long-term stents is also encouraging (13–16). However, there are few reports on endovascular treatment of IVCS in children. Oguzkurt et al. have reported that they placed a 12-mm Wallstent in the left common iliac vein of a 10-year-old boy (17). Although the swelling of the left lower extremity of the boy was significantly improved, the mid- and long-term effects are uncertain.

In the patient presented here, his main clinical manifestation is left lower extremity swelling. CTA and MRI showed the left common iliac vein had become narrow before it entered the right common iliac vein. Next, we performed angiography to confirm the diagnosis. We also found the aorta descended to the right of the spine. This might be the reason for the apparent compression of the left common iliac vein. Considering that the boy was young and had mild symptoms, conservative treatments were applied to this boy mainly including elevation of the affected limb, wearing medical elastic socks, and short-term oral aspirin for anticoagulation. The boy is followed up closely. If the swelling of the left extremity significantly increases, stent placement may need to be considered in the future.

IVCS (May–Thurner syndrome) in children is rarely reported. Here we reported a case of IVCS in a 5-year-old boy. This will help clinicians better learn about and understand IVCS in children.

## Data Availability Statement

The original contributions presented in the study are included in the article/supplementary material, further inquiries can be directed to the corresponding author/s.

## Ethics Statement

The studies involving human participants were reviewed and approved by Ethics Committee of children's Hospital Affiliated to Nanjing Medical University. Written informed consent to participate in this study was provided by the participants' legal guardian/next of kin. Written informed consent was obtained from the individual(s), and minor(s)' legal guardian/next of kin, for the publication of any potentially identifiable images or data included in this article.

## Author Contributions

LJ and FY designed the study and edited the manuscript. JC, XChen, JY, and XCheng cared for the patient and contributed samples collection. YQ and SY revised the paper. All authors have read and approved the final manuscript.

## Conflict of Interest

The authors declare that the research was conducted in the absence of any commercial or financial relationships that could be construed as a potential conflict of interest.
